# Tensile Bond Strength and Retention of Three Types of Ceramic Endocrowns

**DOI:** 10.30476/DENTJODS.2022.92053.1619

**Published:** 2023-03

**Authors:** Anahita Fayyazi, Leila Habibi, Bijan Heidari, Sara Tavakolizadeh

**Affiliations:** 1 Postgraduate Student, Dept. of Prosthodontics, School of Dentistry, Shahid Beheshti University of Medical Sciences, Tehran, Iran; 2 Dept. of Prosthodontics, School of Dentistry, Shahid Beheshti University of Medical Sciences, Tehran, Iran

**Keywords:** Tensile bond Strength, Dental restoration, Ceramics, CAD-CAM, Resin cement

## Abstract

**Statement of the Problem:**

By development of adhesive dentistry and noble mechanical strength of ceramics, reconstruction of posterior teeth with partial coverage restorations such as ceramic endocrowns is possible. Different ceramics may show different mechanical properties which should be investigated.

**Purpose:**

The aim of this experimental *in vitro* study was to compare the tensile bond strength of endocrowns made by CAD-CAM using 3 types of ceramics.

**Materials and Method:**

In this *in vitro* study, 30 fresh extracted human molars were prepared to evaluate the tensile bond strength of endocrowns made by IPS e.max CAD, Vita Suprinity, and Vita Enamic blocks (n=10). The specimens were mounted and endodontically treated. Standard preparations were done with 4.5±0.5 mm intracoronal extensions into the pulp chamber and the restorations were designed and milled by CAD-CAM technique. All specimens were cemented with a dual polymerizing resin cement according to the manufacturer's instructions. The specimens were incubated for 24 hours and then thermocycled for 5000 cycles at 5-55°C and underwent the tensile strength test by universal testing machine (UTM). Shapiro-Wilk and one-way ANOVA test were used to statistically analyzed (α= 0.05).

**Results:**

The highest tensile bond strength values were achieved in IPS e.max CAD (216.39 ±22.67N) and Vita Enamic (216.22±17.72N) followed by Vita Suprinity (211.54±20.01N). There was no significant statistical difference between retention of endocrowns made by CAD-CAM technique among ceramic blocks (*p*= 0.832).

**Conclusion:**

Within the limitation of this study, there was no significant difference between retention of endocrowns made by IPS e.max CAD, Vita Enamic, and Vita Suprinity as ceramic blocks.

## Introduction

Reconstruction of extensively damaged teeth is a common challenging issue in restorative dentistry [ 1 ]. Many factors such as bond strength of an adhesive system, the restorative material thickness, proximity of the modulus of elasticity between the restoration material and tooth structure, and the presence of microleakage affect the longevity of the restoration [ 1 - 2 ].

There are different methods to restore endodontically treated teeth. Direct build-up restorations traditionally include prefabricated post and amalgam core, which can lead to unsatisfactory outcomes like root fracture, microleakage, and material deterioration. Following the development of adhesive systems and glass ionomer cement, the microleakage problem was resolved to a great extent, leaving other problems unchanged [ 3 ].

Usage of cast post and core is another way to stabilize the restoration of endodontically treated teeth [ 4 ]. Studies showed that this treatment modality failure could be due to the mechanical characteristics of tooth structure [ 4 ]. Disadvantages like re-infection of the root canal system, tooth perforation, dependency on root morphology, increased chair-time, and prolonged laboratory procedures, give rise to the prevalent application of adhesive restorations with the pulp chamber as the main element of retention [ 3 ].

The concept of endocrowns was first introduced by Pissis in 1995. Subsequently, Bindl and Mormann have extended this term in 1999. These materials can rehabilitate teeth without adequate ferrule effect by utilizing the macro-retentive of pulp chamber walls and micro-retentive of adhesive cementations. In the meantime, omitting the need for post and core restorations put the endocrowns among conservative treatment options [ 5 ].

Over the past 10 years, computer-aided design/ computer-aided manufacturing (CAD-CAM) technology had extensive advertising that can be used instead of conventional lab techniques [ 6 ]. This method improves the mechanical properties of materials and also eliminates defects of manual procedures [ 7 ].

Dental ceramics are the main categories of materials that can be used with CAD-CAM technology [ 6 ]. These materials are subdivided into three principal subgroups: glass-matrix ceramics, polycrystalline ceramics, and resin-matrix ceramics [ 6 ]. The first two groups include inorganic ceramic materials. The resin-matrix ceramic is made of polymer that contains inorganic compositions such as glass, ceramics, and glass-ceramics [ 8 ]. 

IPS e.max CAD (Ivoclar Vivadent, Schaan, Lichtenstein) is a subgroup of the glass-matrix ceramics which includes approximately 70% volume of lithium disilicate as a crystalline phase. In the manufacturing process, ceramic is cast in lithium orthosilicate (Li_4_SiO_4_) transparent glass to form lithium metasilicate crystals (Li_2_SiO_3_) with an average size of 0.2 to 1.0 µm. This intermediate crystalline phase that called ″blue state″ and can easily be milled in CAD unite. The milled blocks are tempered at 850 °C for 25 min to form the lithium disilicate crystals (Li2Si2O5) [ 9 ].

The first zirconia-reinforced lithium silicate ceramics like Vita Suprinity (Vita Zahnfabrik, Bad Sackingen, Germany) has been introduced for dental CAD-CAM which aims to mix the favorable material characteristics of both lithium silicate ceramic and zirconia (10% by weight). This synthetic material is a subcategory of the glass-matrix ceramics in which adding zirconia result in a round and slightly elongated crystalline structure with an average size of 0.5µm. This phase reinforces the ceramic structure by limiting the crack propagation [ 6 , 8 , 10 ].

To improve mechanical proportions, a new network material in which a porous ceramic is infiltrated by a polymer has been revealed by the Vita Company. The main trait of this resin-matrix ceramic is a fine-grain crystal in the structure without a glassy phase. Vita Enamic (Vita Zahnfabrik, Bad Sackingen, Germany) is a polymer-infiltrated ceramic network (PICN) [ 11 ]. The construction procedure provides a suitable brittleness index for CAD milling unite [ 6 ]. Furthermore, the high modulus elastic, and hardness of this material illustrates similar creep behavior to human enamel, which provides sufficient durability of tooth restorations [ 10 ]. 

Retention of endocrowns is an important factor in the longevity of these restorations. Therefore, this study aimed to evaluate the retention of endocrowns made by CAD-CAM using three types of ceramics including IPS e.max CAD, Vita Suprinity, and Vita Enamic. The null hypothesis of the study was defined, as there is no difference between the tensile bond strength of three tested ceramics.

## Materials and Method

### Preparation of tooth specimen

**Figure 1:**
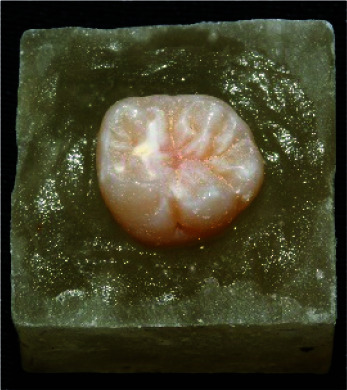
Molar tooth mounted in acrylic resin block

Thirty extracted fresh mandibular molars with completed roots and without cracks, fractures, or decays were cllected and stored in saline solution for 7 days (Figure 1). To standardize the size of the selected teeth, a digital caiper (S235, Sylvac, Switzerland) was used to measure the buccolingual and mesiodistal dimensions of each tooth at the cement-enamel junction (CEJ) Thinner or thicker teeth from 8 to 10mm were excluded from the study [ 2 , 12 ]. Each tooth was mounted in a prefabricated aluminum mold (25×25mm) with the occlusal surface parallel to the horizontal plane, using self-curing acrylic resin (Acropars, Marlic Medical Industries Co., Tehran, Iran). Specimens (n=30) were randomly divided into three groups as Group (1): IPS e.max CAD endocrowns, Group (2): Vita Suprinity endocrowns, and Group (3): Vita Enamic endocrowns. All materials and appliances are described in detail in Table 1. 

**Table 1:** List of materials and their compositions used in the study

Material	Composition	Manufacturer
Lithium disilicate-reinforced vitreous ceramic (IPS e.max CAD)	SiO2, Li2O, K2O, MgO, Al2O3, P2O5 and other oxides	Ivoclar Vivadent AG, Germany
Vitreous ceramic reinforced with lithium silicate and zirconium oxide (Vita Suprinity)	zirconium oxide 8–12%, silicon dioxide 56–64%, lithium oxide 15–21%, various >10%	Vita Zahnfabrik, Bad Sackingen, Germany
Vita Enamic hybrid ceramic (resin infiltrated ceramic network)	Ceramic: silicon dioxide 58%-63%, aluminum oxide 20%-23%, sodium oxide 9%-11%, potassium oxide 4%-6%, boron trioxide 0.5%-2%, zirconia and calcium oxide; Polymer part (25%): UDMA and TEGDMA	Vita Zahnfabrik, Bad Sackingen, Germany
Dual polymerized resin cement (RelyX Unicem 2 Automix)	Base paste: Methacrylate monomers containing phosphoric acid groups, Methacrylate monomers, Silanated fillers, Initiator components, Stabilizers, Rheological additives Catalyst paste: Methacrylate monomers, Alkaline (basic) fillers, Silanated fillers, Initiator components, Stabilizers, Pigments, Rheologicam additives	3 M ESPE, Seefeld, Germany

### Endocrown preparation

Special milling machine (Centroid CNC, milling machine, USA) was used to standardize the preparation of the specimens. The teeth were cut 3-mm above the CEJ. The access cavity was prepared with diamond stone (Dentsply Maillefer, Switzerland) with a total occlusal divergence of 8-10 degrees, the mean depth of the central retention cavity was measured 4.5 ±0.5mm from the decapitation level. The cutting edge was prepared with a diamond wheel (Dentsply Maillefer, Switzerland). The mean remaining thickness of the dentin walls (2.5±0.5mm) was measured by a digital caliper [ 2 , 12 ].

**Figure 2:**
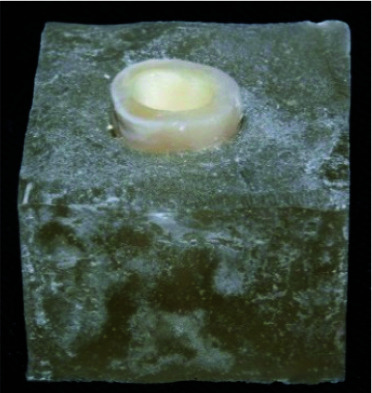
Prepared tooth after mounting in the acrylic mold

All teeth were endodontically treated using rotary files (Dentsply Maillefer, Switzerland) with a continuous irrigation with 5.25% sodium hypochlorite and saline. The obturation technique chosen was the combination of horizontal and vertical compaction technique with gutta-percha (Dentsply Maillefer, Switzerland) and AH Plus (Dentsply, Germany) as the sealer. Then the orifices were filled with restorative glass ionomer (Figure 2).

### Laboratory procedures

**Figure 3:**
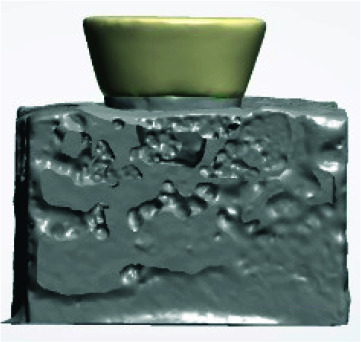
Trapezoid design of restoration

**Figure 4:**
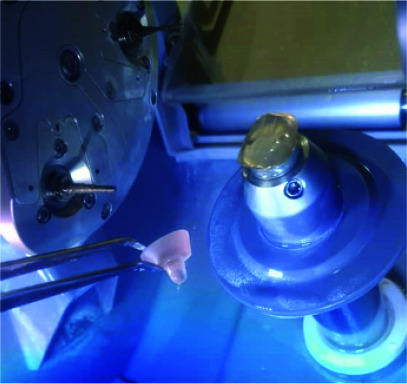
Semi-crystallized Suprinity endocrown before sintering

The prepared teeth were covered with scanning powder (Spotcheck SKD-S2, Magnaflux®, UK) and scanned using a D810 scanner (3Shape, Copenhagen, Denmark). The appropriate design software (2017, 3Shape Dental System) was used to design endocrowns on the virtual model. The cement space was set at 80µ for all samples. Endocrowns were prepared using Sirona inLab MC XL CAD/CAM Milling Machine (Dentsply Sirona Inc., USA) with IPS e.max CAD, Vita Suprinity, and Vita Enamic blocks, subsequently. Ceramic materials are generally brittle. Therefore, endocrowns were designed in a trapezoid shape that can be mounted in acrylic resin blocks for retention tests (Figure 3). The crystallization of semi-crystallized ceramics was performed based on the manufacturer’s process using Vita Vacumat 6000 MP (Vita Zahnfabric, Bad Sackingen, Germany) (Figure 4). All endocrowns were polished using their special polishing kit without additional glazing.

### Bonding procedure

Etching with 5% hydrofluoric acid (IPS Ceramic Etching Gel, Ivoclar Vivadent, Schaan, Liechtenstein) for 20 seconds was done on the tissue surfaces of endocrowns in IPS e.max CAD and Vita Suprinity groups and 60 seconds for Vita Enamic. After etching, each restoration was cleaned in an ultrasonic apparatus for five minutes and then dried with oil-free air spray. A thin layer of silane coupling agent (Prosil; FGM) was applied to the internal walls of the endocrowns for 60 seconds and then air-dried.

**Figure 5:**
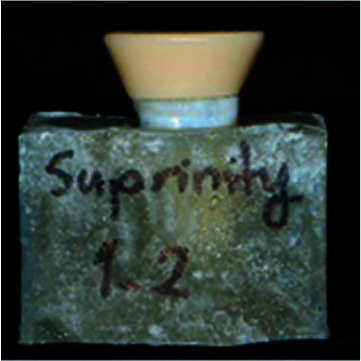
An endocrown restoration after cementation

Self-adhesive resin composite cement (RelyX Unic-em 2 Automix, 3 M ESPE, Seefeld, Germany) with a 1: 1 base-catalyst ratio was mixed to obtain a uniform consistency. The cement was used on the tissue surface of the endocrowns. The restoration was placed on the tooth with a 3 kg weight in a load applicator. The excess cement was removed after 2-3 minutes from the start of the mix. Then the cement was light-activated for 20 seconds. A light-emitting diode curing unit (Demetron A.1, Kerr/Sybron, Orange, CA, USA) with a 12-mm diameter curing light tip and irradiance output of 1000±50mW/cm^2^ was used. The surface-tip distance was 0.5mm (Figure 5). After cementation, all samples were kept in an incubator (Model 2; Precision Scientific Co., Columbus, OH, USA) at 37°C for 24 hours.

### Thermal cycling

**Figure 6:**
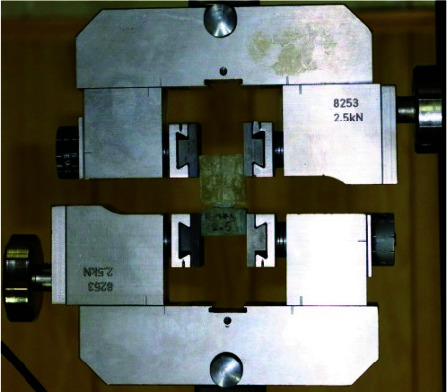
The specimen mounted in a custom made jig in the UTM

The samples were placed in a thermocycling device (TC-300, Vafaei industrial, Iran) for 5 days to simulate the temperature changes and aging process of an oral cavity. The specimens were exposed to 5000 thermal cycles (to simulate 1 year of average human masticatory function), between 5˚C-55˚C, with a dwell time of the 30s and transfer time of 10s (Figure 6). Before testing the tensile bond strength, endocrowns were mounted in the acrylic resin in the same manner as the prepared tooth. The specimens then were mounted on a custom jig and the dislodging force was applied in the perpendicular direction to the occlusal surface of the specimens with the universal testing machine (Zwick, Krefeld, Germany). Each sample was installed separately on the device and the tensile strength test was performed with a crosshead speed of 0.5mm/ min (Figure 3).

Statistical analysis was performed with SPSS 26 (Statistical Package for Scientific Studies, Inc., USA). The data were analyzed by Shapiro-Wilk and one-way ANOVA tests. The significance level was 0.05%.

## Results

**Figure 7:**
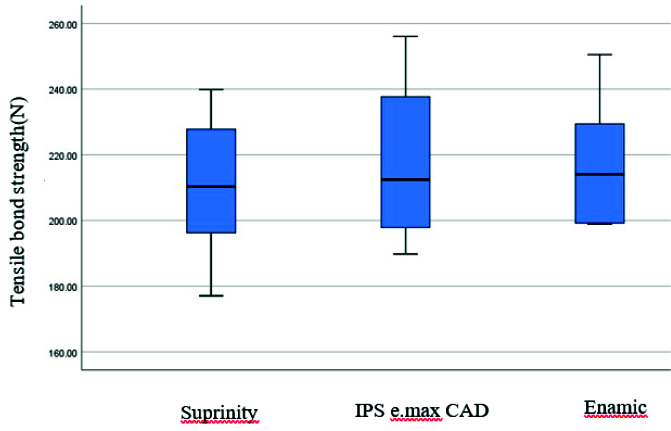
Bar graph of mean tensile bond strength values in Newton for different CAD-CAM materials

The mean and SD values of tensile bond strength and retention for all groups are illustrated in Table 2 and Figure 7.

**Table 2:** Descriptive tensile bond strength values in Newton for different CAD-CAM materials

CAD/CAM materials	Minimum	Maximum	Mean(Std. deviation)
IPS e.max CAD (N=10)	189.75	256.06	216.39(22.67)
Suprinity (N=10)	177.05	239.86	211.54(20.01)
Enamic (N=10)	198.90	250.50	216.22(17.72)

As the distribution of the data proved to be normal by Shapiro-Wilk Test, parametric tests were selected for comparing the data. Based on the results of the one-way analysis of variance in Table 3, there was no significant difference between tensile bond strength of endocrowns made by CAD-CAM technique regarding the ceramic type (*p*= 0.832).

**Table 3:** The one-way analysis of variance (ANOVA) test

	Sum of squares	Degrees of freedom	Average of squares	F	The significance level
Between group	151.774	2	75.887	0.185	0.832
Within group	11058.796	27	409.585		
Sum	11210.570	29			

## Discussion

There is no consensus on the best treatment plan for posterior endodontically treated teeth; residual coronal structure seems to be the most important factor in the long-term prognosis of the restoration [ 5 ]. Although the use of post-core-crown has become a classic method for the reconstruction of severely damaged teeth, this notion has changed since the advent of adhesives in conservative dental treatment [ 5 ].

Resistance to masticatory forces and proper retention is one of the most effective items in the clinical prognosis of conservative restoration [ 12 ]. ‬In the oral environment, restoration is affected by various forces such as compressive, tensile, and shear forces intermittently and frequently. Accumulation of these forces may lead to a failure in the bonding interference of tooth and the restoration in the long term, leading to the loss of retention. ‬‬‬

The purpose of this study was to compare the retention of endocrowns made by CAD-CAM using three types of ceramics; IPS e.max CAD, Vita Suprinity, and Vita Enamic blocks. In the present study, the highest mean tensile bond strength was reported for IPS e.max CAD and Enamic groups and the lowest mean tensile bond strength was seen in the Suprinity group. However, no significant statistical difference was observed between the tensile bond strength of these three groups.

Bellan *et al*. [ 13 ] evaluated the micro-tensile bond strength of CAD-CAM restorative materials to dentin using different adhesive systems. It was declared that polymer infiltrated ceramic blocks (Enamic) had higher bond strength values compared to vitreous ceramic blocks (Suprinity). They mentioned the differences in modulus of elasticity among the restorative blocks as the main factor for their findings. the Enamic modulus of elasticity was measured at 30.1 GPa, which is close to the dentin modulus of elasticity (16-20.3GPa) and lower than the Suprinity modulus of elasticity (70.44GPa) [ 14 - 15 ]. 

Brittle ceramic blocks may initiate crack at adhesive /ceramic interface at lower values than the more resilient ones like Enamic. In addition, after design and milling, Enamic blocks do not need to be placed in a furnace to complete crystallization, which reduces the chair time [ 16 ]. In this study, the average tensile bond strength of endocrowns made by Enamic was higher than Suprinity, but this difference was not statistically significant. 

Zirconia polycrystalline structure is the main reason for the weak bond strength of Y-TZP ceramics [ 17 ]. Since Suprinity ceramic blocks have zirconia in their structure, it was expected that the tensile bond strength of endocrowns made by Suprinity blocks is less than the other tested blocks. In the present study, no significant difference was observed between the tensile bond strength of endocrowns made by IPS e.max CAD, Suprinity, and Enamic. Therefore, the null hypothesis was accepted. One of the reasons can be attributed to the difference in the morphology of the pulp chamber as the retention cavity. Removal of ceramic particles and increasing surface roughness after etching is the cause of micromechanical retention of ceramic blocks. The highest values of tensile bond strength were reported in CAD/CAM blocks after 15-60 seconds of etching with hydrofluoric acid [ 18 - 19 ]. Recent studies have shown that prolonged etching (20 to 120 seconds) does not increase bond strength in lithium disilicate ceramics [ 20 - 21 ]. A total of 30-60 seconds etching is also recommended for polymer-infiltrated ceramics for maximum tensile bond strength [ 22 - 23 ]. In the present study, E.max CAD and Suprinity were etched for 20 seconds and Enamic for 60 seconds. 

In addition, ceramic etching leads to exposure of hydroxyl groups and allows chemical interactions with the Silane coupling agent [ 24 - 25 ]. The use of a silane coupling agent to increase the surface energy and improve retention between resin cement and restorative material has been widely suggested [ 26 ]. Therefore, in this study, silane coupling agent was used for all specimens.

Multi-stage application of total-etch adhesives, and their increased chair time serve as main disadvantages in the bonding procedure. Because of these limitations, self-adhesive resin cements were introduced for decreasing the whole process and shortening the window of contamination [ 27 - 28 ]. Therefore, self-adhesive resin cements are recommended for bonding endocrown restorations according to similar articles and were chosen as a luting agent in the present study [ 29 ].

Jing *et al*. [ 30 ] showed that increasing the occluso-cervical height of the preparation leads to an increase in the tensile bond strength of the restoration. In the present study, the mean of pulp chamber height was considered to be 4.5±0.5mm. There have been some limitations in this study. Differences in pulp chamber morphology could make bias in results. Further studies are needed to investigate the mechanical and adhesive properties of the materials used in and even long-term follow-up sessions in the clinic.

## Conclusion

The present study showed that the use of IPS e.max CAD, Suprinity, and Enamic ceramic blocks to build indirect conservative restorations is promising. However, further studies are needed to investigate the mechanical and adhesive properties of endocrowns in prolonged follow-up sessions.

## Acknowledgements

The authors are grateful to Toos Dental Lab for the laboratory procedures.

## Conflict of Interest

The authors declare that they have no conflict of interest.

## References

[1] Kanat-erturk B, Sariday S, Koseler E, Helvacioglu-Yigit D, Avcu E, Yildiran-Avcu Y ( 2018). Fracture strengths of endocrown restorations fabricated with different preparation depths and CAD-CAM materials. Dent Mater J.

[2] El-Damanhoury HM, Haj-Ali RN, Platt JA ( 2015). Fracture resistance and microleakage of endocrowns utilizing three CAD-CAM blocks. Oper Dent.

[3] Sun J, Ruan W, He J, Lin X, Ci B, Yin S, et al ( 2019). Clinical efficacy of different marginal forms of endocrowns: study protocol for a randomized controlled trail. Trials.

[4] Biacchi GR, Mello B, Basting RT ( 2013). The endocrown: an alternative approach for restoring extensively damaged molars. J Esthet Restor Dent.

[5] Al-Dabbagh RA ( 2021). Survival and success of endocrowns: A systematic review and meta-analysis. J Prosthet Dent.

[6] Hassanzadeh M, Sahebi M, Zarrati S, Payaminia L, Alikhasi M ( 2021). Comparative of evaluation of the internal and marginal adaptation of CAD-CAM endocrowns and crowns fabricated form three different materials. Int J Prosthodont.

[7] Rocca GT, Saratti CM, Poncet A, Feilzer AJ, Krejci I ( 2016). The influence of FRCs reinforcement on marginal adaptation of CAD-CAM composite resin endocrowns after simulated fatigue loading. Odontology.

[8] Gracis S, Thompson VP, Ferencz JL, Silva NR, Bonfante EA ( 2015). A new classification system for all ceramic and ceramic-like restorative materials. Int J Prosthodont.

[9] Bajraktarova-Valjakova E, Korunoska-Stevkovska V, Kapusevska B, Gigovski N, Bajraktarova-Misevska C, Grozdanov A ( 2018). Contemporary dental ceramic material, a review: chemical composition, physical and mechanical properties, indications for use. Open Access Maced J Med Sci.

[10] Sen N, Olcer Us Y ( 2018). Mechanical and optical properties of monolithic CAD-CAM restorative materials. J Prosthet Dent.

[11] He LH, Swain M ( 2011). A novel polymer infiltrated ceramic dental material. Dent Mater.

[12] Zheng Z, He Y, Ruan W, Ling Z, Zheng Ch, Gai Y, et al ( 2021). Biomechanical behavior of endocrown restorations with different CAD-CAM materials: A 3D finite element and in vitro analysis. J Prosthet Dent.

[13] Bellan MC, Cunha P, Tavares JG, Spohr AM, Mota EG ( 2017). Microtensile bond strength of CAD/CAM materials to dentin under different adhesive strategies. Braz Oral Res.

[14] Homaei E, Farhangdoost K, Tsoi JKH, Matinlinna JP, Pow EHN ( 2016). Static and fatigue mechanical behavior of three dental CAD/CAM ceramics. J Mech Behav Biomed Mater.

[15] Coldea A, Swain MV, Thiel N ( 2013). Mechanical properties of polymer-infiltrated-ceramic-network materials. Dent Mater.

[16] El Zohairy AA, De Gee AJ, Mohsen MM, Feilzer AJ ( 2003). Microtensile bond strength testing of luting cements to prefabricated CAD/CAM ceramic and composite blocks. Dent Mater.

[17] Al-Harbi FA, Ayad NM, Khan ZA, Mahrous AA, Morgano SM ( 2016). In vitro shear bond strength of Y-TZP ceramics to different core materials with the use of three primer/resin cement systems. J Prosthet Dent.

[18] ‬Ataol AS, Ergun G ( 2018). Effects of surface treatments on repair bond strength of a new CAD/CAM ZLS glass ceramic and two different types of CAD/CAM ceramics. J Oral Sci.

[19] Tian T, Tsoi JK, Matinlinna JP, Burrow MF ( 2014). Aspects of bonding between resin luting cements and glass ceramic materials. Dent Mater.

[20] Mokhtarpour F, Alaghehmand H, Khafri S ( 2017). Effect of hydrofluoric acid surface treatments on micro-shear bond strength of CAD/CAM ceramics. Electron Physician.

[21] Puppin-Rontani J, Sundfeld D, Costa AR, Correr AB, Puppin-Rontani RM, Borges GA, et al ( 2017). Effect of Hydrofluoric Acid Concentration and Etching Time on Bond Strength to Lithium Disilicate Glass Ceramic. Oper Dent.

[22] ‬Lee Y, Kim JH, Woo JS, Yi YA, Hwang JY, Seo DG ( 2015). Analysis of Self-Adhesive Resin Cement Microshear Bond Strength on Leucite-Reinforced Glass-Ceramic with/without Pure Silane Primer or Universal Adhesive Surface Treatment. Biomed Res Int.

[23] Schwenter J, Schmidli F, Weiger R, Fischer J ( 2016). Adhesive bonding to polymer infiltrated ceramic. Dent Mater.

[24] Lung CY, Matinlinna JP ( 2012). Aspects of silane coupling agents and surface conditioning in dentistry: an overview. Dent Mater J.

[25] Özcan M, Volpato CA ( 2015). Surface Conditioning Protocol for the Adhesion of Resin-based Materials to Glassy Matrix Ceramics: How to Condition and Why?. J Adhes Dent.

[26] ‬Kawaguchi A, Matsumoto M, Higashi M, Miura J, Minamino T, Kabetani T, et al ( 2016). Bonding effectiveness of self-adhesive and conventional-type adhesive resin cements to CAD/CAM resin blocks. Part 2: Effect of ultrasonic and acid cleaning. Dent Mater J.

[27] Burgess JO, Ghuman T, Cakir D ( 2010). Self-adhesive resin cements. J Esthet Restor Dent.

[28] Ferracane JL, Stansbury JW, Burke FJ ( 2011). Self-adhesive resin cements-chemistry, properties and clinical considerations. J Oral Rehabil.

[29] ‬Fages M, Bennasar B ( 2013). The endocrown: a different type of all-ceramic reconstruction for molars. J Can Dent Assoc.

[30] ‬Jing L, Chen JW, Roggenkamp C, Suprono MS ( 2019). Effect of crown preparation height on retention of a prefabricated primary posterior zirconia crown. Pediatr Dent.

